# Cerebral Lactate Concentration in Neonatal Hypoxic-Ischemic Encephalopathy: In Relation to Time, Characteristic of Injury, and Serum Lactate Concentration

**DOI:** 10.3389/fneur.2018.00293

**Published:** 2018-05-11

**Authors:** Tai-Wei Wu, Benita Tamrazi, Kai-Hsiang Hsu, Eugenia Ho, Aaron J. Reitman, Matthew Borzage, Stefan Blüml, Jessica L. Wisnowski

**Affiliations:** ^1^Department of Pediatrics, Keck School of Medicine, Fetal and Neonatal Institute, Division of Neonatology, Children’s Hospital Los Angeles, University of Southern California, Los Angeles, CA, United States; ^2^Department of Radiology, Children’s Hospital Los Angeles, Keck School of Medicine, University of Southern California, Los Angeles, CA, United States; ^3^Division of Neonatology, Department of Pediatrics, Chang Gung Memorial Hospital Linkou Branch, Taoyuan, Taiwan; ^4^Department of Neurology, Children’s Hospital Los Angeles, Keck School of Medicine, University of Southern California, Los Angeles, CA, United States; ^5^Division of Neonatology, Department of Pediatrics, LAC + USC Medical Center, Keck School of Medicine, University of Southern California, Los Angeles, CA, United States; ^6^Rudi Schulte Research Institute, Santa Barbara, CA, United States

**Keywords:** lactate, cerebral lactate, magnetic resonance spectroscopy, neonatal asphyxia, hypoxic-ischemic encephalopathy

## Abstract

**Background:**

Cerebral lactate concentration can remain detectable in neonatal hypoxic-ischemic encephalopathy (HIE) after hemodynamic stability. The temporal resolution of regional cerebral lactate concentration in relation to the severity or area of injury is unclear. Furthermore, the interplay between serum and cerebral lactate in neonatal HIE has not been well defined. The study aims to describe cerebral lactate concentration in neonatal HIE in relation to time, injury, and serum lactate.

**Design/methods:**

Fifty-two newborns with HIE undergoing therapeutic hypothermia (TH) were enrolled. Magnetic resonance imaging and spectroscopy (MRI + MR spectroscopy) were performed during and after TH at 54.6 ± 15.0 and 156 ± 57.6 h of life, respectively. Severity and predominant pattern of injury was scored radiographically. Single-voxel ^1^H MR spectra were acquired using short-echo (35 ms) PRESS sequence localized to the basal ganglia (BG), thalamus (Thal), gray matter (GM), and white matter. Cerebral lactate concentration was quantified by LCModel software. Serum and cerebral lactate concentrations were plotted based on age at time of measurement. Multiple comparisons of regional cerebral lactate concentration based on severity and predominant pattern of injury were performed. Spearman’s Rho was computed to determine correlation between serum lactate and cerebral lactate concentration at the respective regions of interest.

**Results:**

Overall, serum lactate concentration decreased over time. Cerebral lactate concentration remained low for less severe injury and decreased over time for more severe injury. Cerebral lactate remained detectable even after TH. During TH, there was a significant higher concentration of cerebral lactate at the areas of injury and also when injury was more severe. However, these differences were no longer observed after TH. There was a weak correlation between serum lactate and cerebral lactate concentration at the BG (*r*_s_ = 0.3, *p* = 0.04) and Thal (*r*_s_ = 0.35, *p* = 0.02). However, in infants with moderate–severe brain injury, a very strong correlation exists between serum lactate and cerebral lactate concentration at the BG (*r*_s_ = 0.7, *p* = 0.03), Thal (*r*_s_ = 0.9 *p* = 0.001), and GM (*r*_s_ = 0.6, *p* = 0.04) regions.

**Conclusion:**

Cerebral lactate is most significantly different between regions and severity of injury during TH. There is a moderate correlation between serum and cerebral lactate concentration measured in the deep gray nuclei during TH. Differences in injury and altered regional cerebral metabolism may account for these differences.

## Introduction

Neonatal hypoxic-ischemic encephalopathy (HIE) affects about 1–6 per 1,000 live births (1). Therapeutic hypothermia (TH) is the first empirically supported therapy for neuroprotection in neonates with HIE. Still, even with TH, 40–50% continue to suffer adverse outcomes (2, 3). The pathophysiology of HIE involves a complex cascade of cellular and molecular processes, which centers on a decline in oxidative metabolism (4, 5). The goal of TH is to mitigate the ischemic cascade and restore energy homeostasis.

Energy metabolism can be monitored *in vivo* in brain tissue by way of MR spectroscopy (MRS). Serial MRS has been employed extensively in laboratory studies to interrogate temporal changes in the concentrations of key metabolites, including lactate, a byproduct of anaerobic metabolism and a biomarker for impaired oxidative metabolism (6). However, MRS is technically demanding in the clinical setting and even more so when performed during TH (7). For this reason, very little is known about the temporal evolution of cerebral lactate concentrations during and after TH in neonates with HIE. Furthermore, neuroimaging studies have established two different patterns of brain injury in neonates with HIE: a central pattern, characterized by injury to the deep gray [thalamus, basal ganglia (BG)] and perirolandic cortex, and a peripheral pattern, characterized by injury to the parasagittal cortex and underlying white matter (WM) (8, 9). It is not known whether regional cerebral lactate concentrations mirror the pattern or severity of brain injury during and/or after TH.

Finally, it should be noted that cerebral lactate may be locally produced in brain tissue or transported into and out of the brain *via* the blood–brain barrier (10). Moreover, the neonatal brain is characterized by a high capacity for lactate transport into and out of the brain (11, 12). Elevations in serum lactate are ubiquitous at birth in neonates with HIE and may even transiently increase before normalizing in the hours to days that follow (13, 14). Furthermore, prolonged elevation in serum lactate is associated with severe encephalopathy and seizure burden (15). Considering the high capacity for lactate transport into the neonatal brain and the underlying association between serum lactate and brain injury, it is important to assess the relation between serum lactate and cerebral lactate in neonates with HIE.

The objectives of this descriptive study were: (1) to determine the temporal resolution of serum and cerebral lactate concentrations in neonates with HIE; (2) to determine whether regional cerebral lactate concentration differs with respect to injury severity or the predominant pattern of brain injury (normal, peripheral/watershed, central/deep gray); and (3) to explore the relationship between serum lactate and cerebral lactate in neonates with HIE undergoing TH. We hypothesize that cerebral lactate is elevated specifically in regions of injury and decreases with time after injury. Furthermore, we hypothesize that serum lactate is more strongly associated with cerebral lactate concentrations in regions of high metabolic demand and marked susceptibility to acute injury in neonates with HIE.

## Materials and Methods

Infants diagnosed with HIE who were admitted to the Children’s Hospital Los Angeles Newborn and Infant Critical Care unit for TH were prospectively enrolled into an observational research study from April 2012 to July 2017. After obtaining written parental permission, patients underwent a research MRI during TH, as well as a standard clinical MRI after re-warming. Serum lactate concentrations and other clinical data were abstracted from medical records. Neurodevelopmental follow-up is ongoing. The study was approved by the Institutional Review Board at Children’s Hospital Los Angeles.

### Patient Sample

Inclusion criteria for TH at our institution is as follows: gestational age of at least 35 weeks, birthweight >1,800 g, admitted within 6 h of age with the history of cord blood gas or first hour blood gas of a pH of ≤7.0 or a base deficit of ≥16 mmol/L. In cases where pH fell between 7.01 and 7.15 or the base deficit was between 10 and 15.9 mmol/L, the following additional criteria must be met: (1) history of an acute perinatal event and (2) a 10-min Apgar score of ≤5 or the need for assisted ventilation at birth for more than a 10-min duration. Patients who met the above criteria underwent TH if they had moderate or severe encephalopathy based on the modified Sarnat score (16), or if they presented with clinical seizures. Whole-body TH was initiated within 6 h of age and a target rectal temperature (33.5–34.5°C) was maintained for 72 h by Cincinnati Sub-Zero Blanketrol III (Gentherm, Cincinnati, OH, USA). At the end of 72 h of whole-body TH, the infants were actively rewarmed to normothermia by incremental increase of rectal temperature at a velocity of 0.5°C per hour up to 36.5 C.

Infants with congenital anomalies, metabolic disorders, early onset sepsis, or perinatal stroke have been retrospectively excluded from all studies from this cohort. Furthermore, we excluded infants who were clinically unstable to undergo research MRI during TH.

### Serum Lactate Collection

Serum lactate concentration was measured every 6–8 h from initiation of TH until completion of rewarming, based on unit TH protocol. Blood samples were obtained from free flowing arterial lines and lactate concentration analyzed as point of care testing using the Epoc^®^ blood analysis system (Alere Inc., Waltham, MA, USA), which has a measurement range of 2.7–180.2 mg/dL. When serum lactate concentrations were found to be greater than the measurable range of 180.2 mg/dL, the value of 180 mg/dL was used for data analysis. For the final analyses relating serum lactate to brain lactate, we focused on serum lactate measured at two time points: on admission (Lactate 1) and at time of first MRI scan (Lactate 2). Mean time-lapse between Lactate 2 sampling and MRS acquisition was 2.9 ± 2.0 h.

### Magnetic Resonance Imaging and Spectroscopy

All patients underwent MRI during TH and after re-warming. TH was maintained for the duration of transport to the MRI utilizing an external battery pack (7). During the MRI, extension tubing was passed through the waveguide and used to connect the MRI-compatible cooling blanket to the Blanketrol, which remain docked in the MRI control room. Rectal temperature was monitored using an MRI-compatible temperature probe (Philips Medical, Best, The Netherlands) and blanket temperature was manually adjusted to maintain core body temperature within therapeutic range (33.5 ± 0.5°C). There were no adverse events associated with transport or MRI.

As standard clinical practice, a series of standardized MR images (T1-, T2-, and diffusion-weighted) were acquired and reviewed by a board-certified pediatric neuroradiologist for brain injury (see below). MRS data were acquired using a single-voxel point-resolved spectroscopy sequence (PRESS; TE 35 ms, TR 2,000 ms, 128 signal averages, voxel size ~3 cm^3^), localized to the right BG, left thalamus, medial cortical gray matter (GM), and left parietal WM based on axial, sagittal, and coronal T2-weighed images (note that the WM voxel was added after the 10th neonate). The regions of interest were selected *a priori* because they are known areas of vulnerability to hypoxia-ischemia in neonates and corresponded to the central (thalamus/BG/perirolandic cortex) and peripheral/watershed (WM) patterns of brain injury in neonatal HIE (9).

Furthermore, the rationality for the left thalamus and right BG stemmed from our desire to focus on the dominant hemisphere with regard to language and motor functions (left THAL) while also obtaining data from both hemispheres (right BG). Lactate was quantitated from each MRS voxel using LCModel (V6.3-1L) (17), consistent with prior studies in our laboratory (18). For quantitation, the unsuppressed water signal was used as a concentration reference, with tissue water content estimated at a standardized value (86%, i.e., 47.8M) based on published reference data (19). To ensure reliability, spectra of poor quality were excluded *a priori* based on stringent thresholds for line width (<0.05 ppm, i.e., <6.4 Hz) and signal to noise ratio (≥10). Spectra were not excluded based on Cramer–Rao lower bound as this would have biased results toward higher lactate concentrations.

### MRI Injury Classification

A pediatric neuroradiologist (Benita Tamrazi) blinded to clinical course and outcome of the subjects, classified patients with regard to the degree of injury and predominant pattern as observed on the post-cooling MRI, using a previously described scoring system (9). This system relies on acute and subacute signal abnormalities in the BG/thalamus (BG-T) region (score 0–4) and watershed region (score 0–5). Based on the scores, each patient was classified with regard to: (1) pattern of injury (normal, BG-T, or watershed pattern) and (2) severity of injury [normal to mild (nml/mild) or moderate to severe (mod/severe)]. The post-cooling MRI was scored instead of the on-cooling MRI in order to ensure highest sensitivity to detection of injury, as diffusion changes may not become fully apparent during the first 24–48 h of injury (20, 21).

### Data Analysis

Statistical analysis was performed using GraphPad Prism software (GraphPad Software, Inc., La Jolla, CA, USA). Data normality was tested using the D’Agostino & Pearson omnibus normality test. Data are presented as mean ± SD or median (interquartile range, IQR) depending on normality. Both cerebral and serum lactate concentration were plotted according to age at time of scan. Paired *t*-test or non-parametric Wilcoxon matched-pairs signed rank test was used to compare respective regional cerebral lactate concentration during vs. after TH. In order to maintain a fair comparison, we excluded cerebral lactate values if the second MRS was performed after 7 days of age. One-way ANOVA or Kruskal–Wallis test with Tukey’s or Dunn’s multiple comparisons test was used to compare regional cerebral lactate concentrations in nml-mild vs. moderate–severe brain injury and in BG-T vs. watershed pattern of injury. Non-parametric Spearman rank-order correlation was computed to assess the relationship between serum and regional cerebral lactate.

## Results

### Patient Characteristics

From April 2012 to July 2017, 52 infants were enrolled into the prospective study and each underwent research MRS during and after TH. Four patients were excluded from analysis due to congenital anomaly (1), gestational age at birth <35 weeks (1), and poor quality on MR spectra (2). Forty-eight subjects (29 male) with mean gestational age of 39 ± 1.8 weeks and birth weight of 3.3 ± 0.6 kg were included in data analysis. The mean age at which the first (during TH) and second MRI (after TH) were performed was 54.6 ± 15 and 156 ± 57.6 h of life, respectively. Based on modified Sarnat exam (16) on admission, 39 had moderate and 9 had severe encephalopathy, respectively. Based on radiographic scoring of injury severity, 37 were normal to mild (nml/mild) and 11 were moderate to severe (mod/severe). Of note, 7 out of the 9 infants with severe encephalopathy based on Sarnat exam had mod–severe injury on MRI. With regard to pattern of injury: 28 were normal, 8 were BG-Thal pattern, and 12 were watershed pattern. Thirteen infants received dopamine or dobutamine infusion during the first 3 days of life. Only two patients received vasopressor–inotrope infusion at doses ≥10 g/kg/min. None received more than one form of vasopressor–inotrope. Markers of other end-organ injury included the highest value of creatinine, aspartate aminotransferase (AST), and alanine aminotransferase (ALT) within the first 72 h of postnatal life. Median (IQR) creatinine was 0.82 (0.7–1.1) mg/dL, AST 141.5 (87.3–282.3) U/L, and ALT 49 (34–105) U/L, respectively. Only two infants had creatinine level >1.5. Twenty out of the 48 infants had some degree of hepatic dysfunction, defined as AST > 200 U/L and/or ALT > 100 U/L. Of the 48 patients, two died and two required G-tube feedings at discharge.

### Temporal Resolution of Serum Lactate

**Figure 1:**
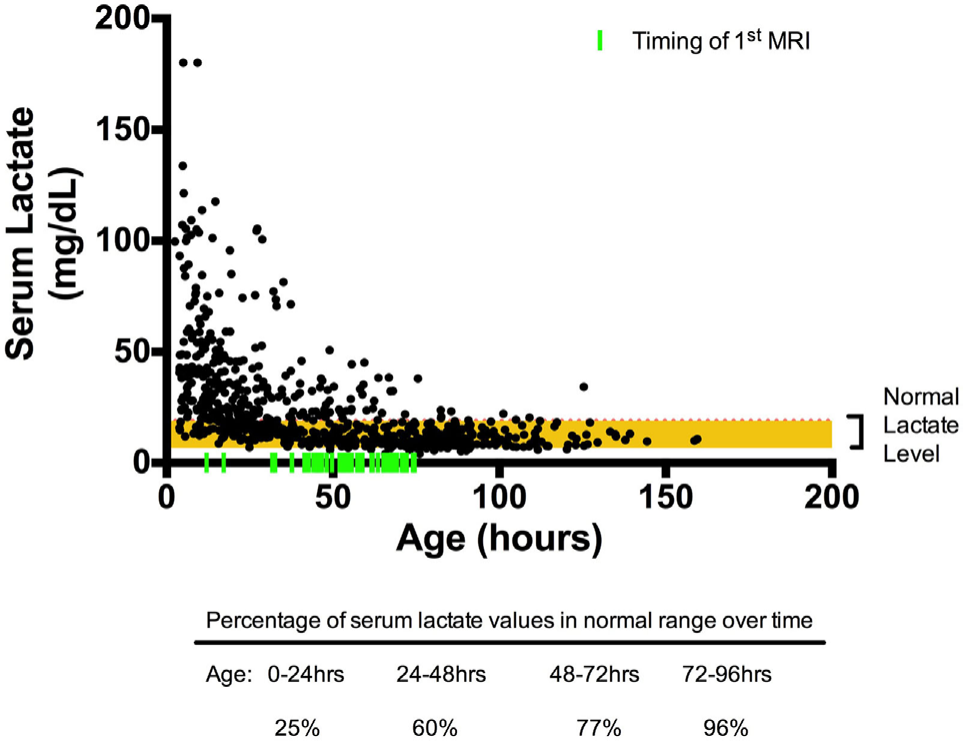
Scatter plot of serum lactate measured over time. All serum lactate values in black circles were plotted over time (*n* = 780) with timing of first MRI indicated by fluorescent green vertical line. The red horizontal line marks the upper limit of normal serum lactate level (19 mg/dL). By 72–96 h of life, 96% percent of the serum lactate values are within reference range.

Overall, 720 serum lactate measurements were obtained for our cohort. The median age at which serum Lactate 1 was obtained was 5.02 (range: 4.1–6.5) hours of life. Serum lactate peaked on or near admission (0–8 h) and trended toward normal over time, as evidenced by the increasing percentages of normal (defined as <19 mg/dL) serum lactate concentrations: 24.6% at 0–24 h; 59.5 at 24–48 h; 77.2% at 48–72 h, and 95.9% at 72–96 h, Figure 1.

### Temporal Resolution of Cerebral Lactate

**Figure 2:**
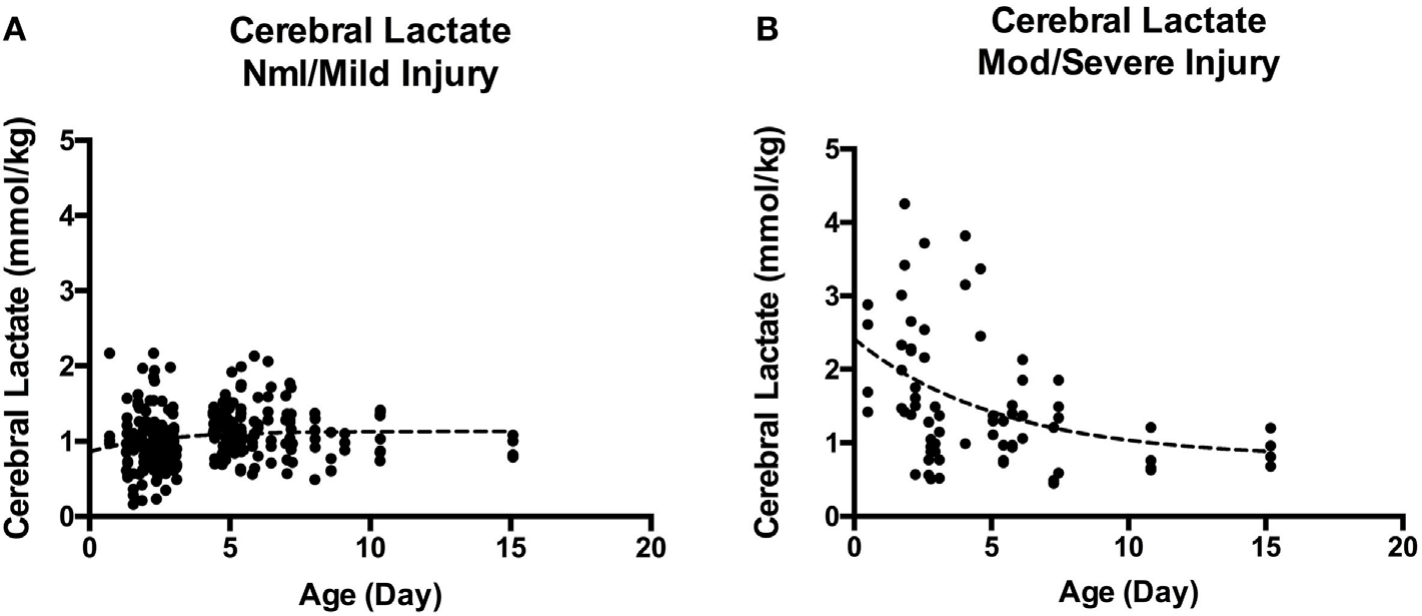
**(A)** Cerebral lactate over time in normal/mild injury. **(B)** Cerebral lactate over time in moderate-severe injury. A marked difference between cerebral lactate clearance in infants with nml/mild injury **(A)** vs. infants with mod/severe injury **(B)**. Although by 2 weeks of life, cerebral lactate trends toward 1 mmol/kg, it remains detectable.

Overall, cerebral lactate concentration was not significantly different *during* vs. *after* TH [1.05 (0.85–1.41) vs. 1.16 (0.94–1.37) mmol/kg, *p* = 0.16]. When examining the cerebral lactate concentration in respective regions (i.e., BG lactate during TH vs. BG lactate after TH) during and after TH, there was also no significant difference. However, when cohort is dichotomized to severity of injury, there was a significant difference in cerebral lactate during vs. after TH. For the nml/mild injury, there was a small, albeit significant *increase* in cerebral lactate concentration from during TH to after TH [1.00 (0.83–1.2) vs. 1.20 (0.94–1.33) mmol/kg, *p* = 0.005]. By contrast, for the mod/severe group, there was a small, but significant *decrease* in cerebral lactate concentration [1.49 (1.0–2.54) vs. 1.3 (0.97–1.9), *p* = 0.028]. In general, as shown in Figure 2, cerebral lactate remained low, across the first 2 weeks of life in the nml/mild group (Figure 2A); however, cerebral lactate was markedly elevated and then, on average, decreased over time among the mod/severe group (Figure 2B).

### Regional Cerebral Lactate in Relation to Severity and Pattern of Injury

**Figure 3:**
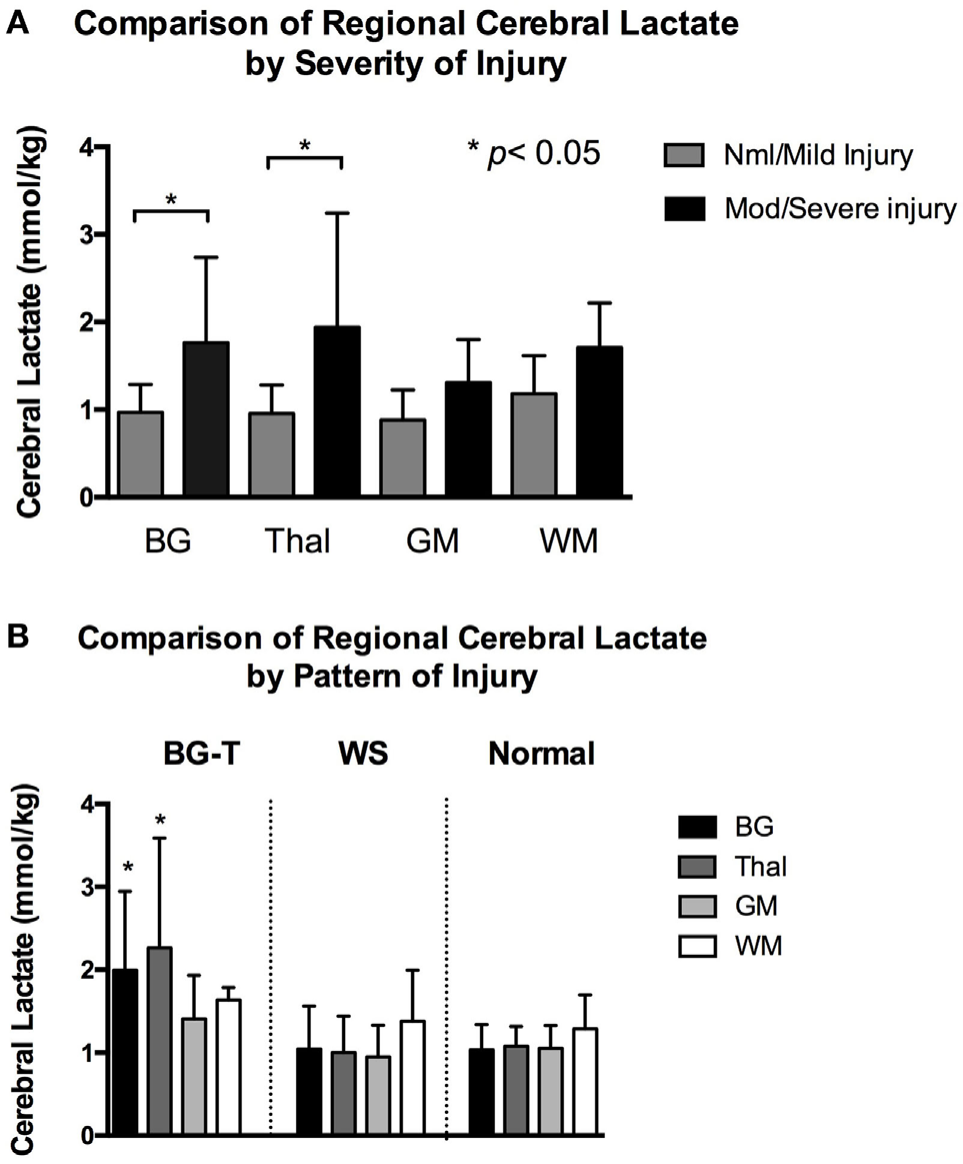
**(A)** Mean regional cerebral lactate concentration during hypothermia in relation to severity of injury. Compared to infants with normal/mild injury, infants with moderate/severe had significantly higher cerebral lactate concentration at the basal ganglia (BG) and thalamus (Thal) regions (denoted by *p < 0.001). There was no significant difference in regional cerebral lactate concentration among the normal/mild injury group. There was also no significant difference in regional cerebral lactate concentration among the moderate/severe injury group. Gray color bars represent normal/mild injury and black bars represent moderate/severe injury. Error bars represent SD. **(B)** Mean regional cerebral lactate concentration during hypothermia in relation to pattern of injury. Infants with predominantly BG-Thal pattern of brain injury had significantly higher cerebral lactate concentration at regions BG and Thal (denoted by *p < 0.05), compared to infants with watershed or normal pattern of injury. Black bars represent cerebral lactate concentration measured at BG. Dark gray bars represent Thal. Light gray bars represent gray matter (GM). White bars represent white matter (WM). Error bars represent SD.

There were no significant differences among regional cerebral lactate concentrate in the nml/mild injury group (all adjusted *p* > 0.3). Similarly, there was no significant difference among regional cerebral lactate concentration in the mod/severe group (all adjusted *p* > 0.4). However, when comparing regional cerebral lactate concentration between severity groups *during* TH, we found that the mod/severe group had significantly higher lactate concentration than the nml/mild group at the BG (1.8 ± 1.0 vs. 1.0 ± 0.3, *p* < 0.001) and Thal (1.9 ± 1.3 vs. 1.0 ± 0.3, *p* < 0.001), Figure 3A.

When examining cerebral lactate levels in regards to pattern of injury (defined radiographically as normal pattern, BG-T pattern, and WS pattern), significantly higher lactate levels were found in the BG and Thal regions in the BG-T pattern (BG 2.0 ± 1.0 and Thal 2.3 ± 1.3 mmol/kg) compared to respective regions in the WS (BG 1.0 ± 0.5 and Thal 1.0 ± 0.4 mmol/kg) and normal pattern (BG 1.0 ± 0.3 and Thal 1.1 ± 0.2 mmol/kg), all adjusted *p* < 0.005 (Figure 3B). In short, significant differences in lactate levels were only detected at the BG and Thal regions when comparing by severity *or* predominant pattern of injury *during* TH. In contrast, *after TH*, there was no significant difference in cerebral lactate concentration at *all* brain regions when comparing infants with regard to injury severity (i.e., nml/mild vs. mod/severe injury, all *p* > 0.06) or pattern (i.e., BG-T vs. WS pattern of injury, all *p* > 0.1).

### Serum Lactate and Cerebral Lactate Correlation

**Figure 4:**
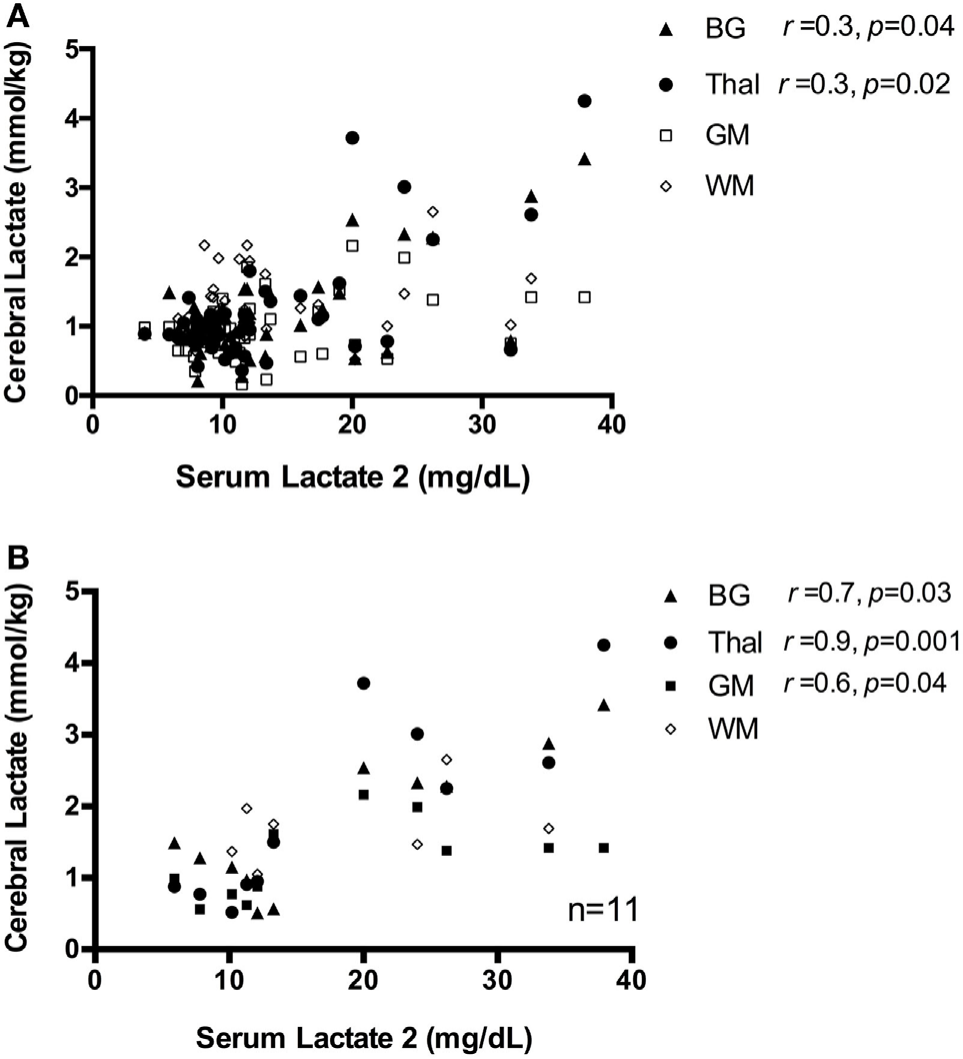
**(A)** Scatter plot of serum Lactate 2 vs. cerebral lactate, all patients. There is a weak correlation between serum Lactate 2 and cerebral lactate concentration, only at regions basal ganglia (BG) and Thal, shown as closed triangle and closed circle, respectively. There is no significant correlation between serum lactate and cerebral lactate at regions gray matter (GM) and white matter (WM), denoted as open square and diamond in the graph. **(B)** Scatter plot of serum Lactate 2 vs. cerebral lactate in infants with moderate–severe injury. For subjects with moderate–severe injury, there is a strong correlation between serum Lactate 2 and cerebral lactate at the BG, Thal, and GM region, which are denoted here in closed triangle, closed circle, and closed square, respectively. There is no significant correlation between serum lactate and WM lactate, denoted as open diamond in the graph.

Across the full sample, there was no significant correlation between serum Lactate 1 and cerebral lactate in either the BG, GM, or WM regions (*p* > 0.05); however, there was a weak positive association in the Thal (*r*_s_ = 0.4, *p* = 0.006). Likewise, there was a weak positive association between serum Lactate 2 and cerebral lactate concentration at the BG (*r*_s_ = 0.3, *p* = 0.04) and Thal (*r*_s_ = 0.35, *p* = 0.02), but no correlation between Lactate 2 and cerebral lactate in the GM and WM regions (both *p* > 0.05), Figure 4A. When limiting our analysis to infants with moderate–severe injury (*n* = 11), the correlation between serum Lactate 2 and cerebral lactate concentration at the BG, Thal, and GM regions became strong and significant (*r*_s_ = 0.7, *p* = 0.03, *r*_s_ = 0.9, *p* = 0.001, and *r*_s_ = 0.6, *p* = 0.04, respectively), Figure 4B.

## Discussion

In this prospective observational study of 48 infants with HIE, we investigated the changes in serum and cerebral lactate concentration over time, during and after TH. We also described the differences in cerebral lactate concentration in relation to severity and predominant pattern of injury. Finally, we examined the relationship between serum lactate and cerebral lactate concentrations.

As expected, serum lactate was elevated on admission and then normalized over time. By contrast, we did not observe a significant effect of time on cerebral lactate measures (during TH vs. after re-warming), when considered across the entire sample. However, there were markedly different trends in cerebral lactate concentration among the neonates with nml/mild MRIs as compared to those with mod/severe injury. Specifically, cerebral lactate concentration increased slightly after TH for the nml/mild subgroup, but decreased slightly after TH for the mod/severe subgroup. There was also a significant difference in regional cerebral lactate concentration relative to the severity and pattern of injury during TH. These significant differences, however, disappeared after TH. Serum Lactate 2 was modestly associated with BG and Thal lactate; the association became stronger in the subgroup of infants with mod/severe injury.

Lactate is generated by way of redox conversion of pyruvate by lactate dehydrogenase, which is necessary to generate ATP and NAD+ in the setting of low oxygen or insufficient mitochondrial metabolism (22). As such, serum lactate is often interpreted clinically as a surrogate marker for tissue hypoxia and/or ischemia (23, 24). As expected, in this cohort, serum lactate was markedly elevated immediately after birth and then trended toward normal. However, it is notable that by day 3 and 4 of life, 23 and 5% of the sample still demonstrated elevated lactate concentrations (≥20 mg/dL), respectively. This persistent elevation in serum lactate could be attributed to hemodynamic instability during the rewarming phase of TH (13); however, none of the infants remained on vasopressor inotropes during that period. Instead, the observation of a persistently and mildly abnormal concentrations of serum lactate even after restoration of adequate organ perfusion and oxygenation is more likely to be secondary to other mechanisms (25, 26). One possibility is that impaired cellular energy metabolism in injured organs led to an ongoing net flux of lactate into the bloodstream from injured tissues. Another possibility is that stress-induced epinephrine secretion, which stimulates Na-K ATPase and thereby can induce an increase in aerobic glycolysis in the skeletal muscle (26), contributed to elevated serum lactate. Finally, a mismatch between increased astrocytic production of lactate and reduced uptake from neurons may also contribute to elevated extracellular lactate in brain injury (27). Further research is needed to better elucidate the complex interplay between lactate production, uptake, oxidation across the CNS, systemic circulation, and other end organs.

The temporal evolution of cerebral lactate differed by injury severity. The nml/mild injury cohort demonstrated a small but significant increase in cerebral lactate concentration from TH to rewarming. It is important to note that the cerebral lactate concentration for this cohort was relatively low to start with [1.00 (0.83–1.2) mmol/kg]; thus, the clinical significance of this increase is unknown. Nevertheless, this finding is consistent with data from animal models whereby TH attenuates the anaerobic metabolism of glucose to lactate (28).

In contrast, cerebral lactate concentrations were significantly elevated during TH in the mod/severe group (range: 1.5–5 mmol/kg), which then decreased slightly from TH to after re-warming. The initial elevation in cerebral lactate during TH is likely related to neuronal injury, reflecting either a failure in neuronal mitochondrial metabolism and concomitant increase in anaerobic metabolism or an uncoupling of glial-neuronal shuttling (aerobic glycolysis) and a concomitant increase in extracellular lactate (27). However, given the high capacity for lactate transport into and out of the newborn brain, it is possible that over time, excessive lactate is transported out of the brain (29) if not otherwise metabolized (25, 30).

**Figure 5:**
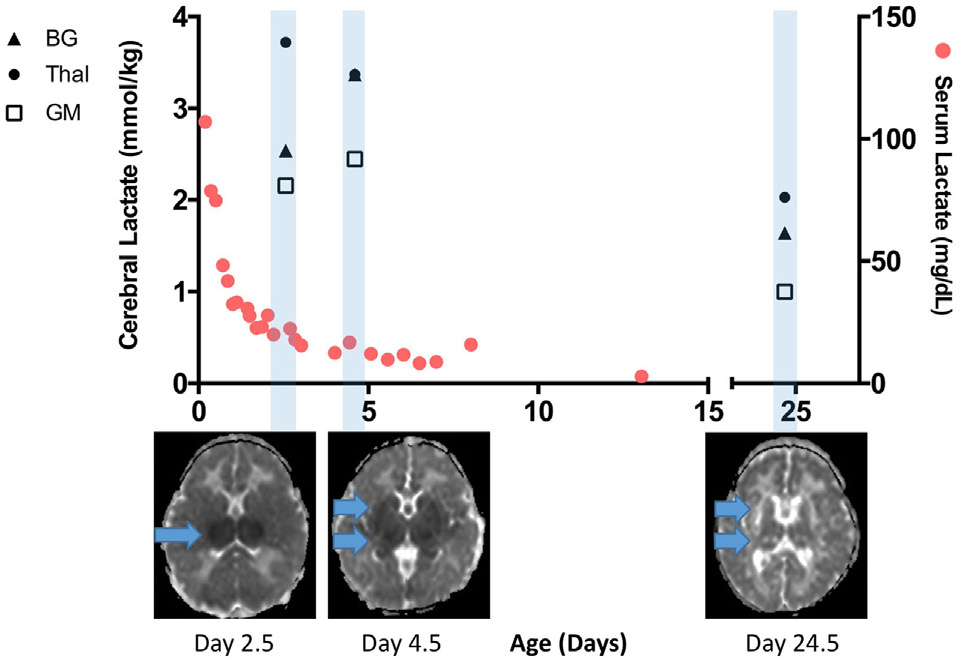
Time-resolution of serum and cerebral lactate in an infant with severe brain injury without any significant end organ injury. Serum lactate rapidly normalized and was <20 mg/dL when the first cerebral lactate was markedly elevated. A repeat scan performed at more than 3 weeks of age reveals lower but still elevated cerebral lactate, especially in the basal ganglia (BG) and thalamus (Thal) regions. Apparent diffusion coefficient map below the *x*-axis reveals progression of injury from the early scan during therapeutic hypothermia (TH) to post-TH, and then chronic injury seen on day 24.5. Cerebral lactate concentration at the BG, Thal, and gray matter (GM) region are denoted here in closed triangle, closed circle, and open square, respectively.

To illustrate the interplay between serum lactate and cerebral lactate over time in the setting of severe HIE and BGT brain injury, we provide a time–resolution plot from one of our patients in (Figure 5). Briefly, this infant was born following uterine rupture and presented with severe encephalopathy on exam without laboratory evidence of kidney injury or hepatic dysfunction. Serum lactate was markedly elevated early but normalized over time. Lactate in the thalamus was markedly elevated during TH, and then decreased slightly after re-warming. By contrast, BG and GM lactate were moderately elevated during TH and then slightly increased after re-warming. Most notably, although cerebral lactate levels on day 24 were lower than during TH, they remained elevated. Given that this patient lacked evidence of any severe extracerebral end-organ injury, the cerebral lactate levels can be attributed to evolving brain injury. This patient died at 5 months of age.

Cerebral lactate concentrations were also higher in accordance to the radiographic scoring of injury, which was determined by a pediatric neuroradiologist blinded to both MRS findings and clinical characteristics. Cerebral lactate concentrations were significantly higher in the BG and Thal regions of infants with moderate–severe injury compared to normal–mild injury. Likewise, cerebral lactate was significantly higher in the BG and Thal regions in neonates with a predominantly BG-T pattern of injury.

Finally, we observed modest associations between serum lactate and cerebral lactate measured in the deep gray nuclei. It has been widely hypothesized that acute, near-total asphyxia events are associated with injury to the thalamus, BG, and perirolandic cortex while partial prolonged events are associated with injury to the parasagittal cortex and underlying WM (31, 32). In line with this, we observed a modest association between serum Lactate 1 and thalamic lactate. Furthermore, we observed associations between serum Lactate 2 and lactate levels in the thalamus and BG. This not only supports the hypothesis that serum lactate, a marker of global hypoxia-ischemia, is most closely associated with injury to the deep gray nuclei, but also suggests that persistent elevations in serum lactate may arise, at least in part, from ongoing flux of lactate from the CNS into the bloodstream.

In the pre-therapeutic hypothermia era, Hanrahan et al. examined cerebral lactate by ^1^H MRS and found that lactate in the BG persisted beyond 1 month after birth in those with poor outcome, while no lactate was detected in infants with normal development or in normal controls (33). We observed a similar pattern, albeit, an earlier decline in cerebral lactate among neonates with moderate to severe injury. Importantly, elevated cerebral lactate appears despite an increase in cerebral blood flow during the subacute phase of injury (34, 35) as well as adult stroke (36). This implies that the initial cerebral lactate peak may occur during the acute phase of injury due to anaerobic metabolism, while the persistence of increased cerebral lactate may be the result of a perturbed cerebral energy metabolism, even under aerobic conditions.

The finding that serum Lactate 2 was more strongly associated with cerebral lactate in the mod/severe injury group than the sample as a whole may be a reflection of the clinical heterogeneity of this cohort. As shown in Figure 4B, this association not only reflects infants with high serum and cerebral lactate but also infants with relatively *low* serum and cerebral lactate. It is unclear if the infants with relatively low serum and cerebral lactate concentration suffered a more subacute injury or if the injury timing was weeks prior to birth. This cohort of mod/severe injury reflects the myriad of injury patterns that is often seen clinically, where severe encephalopathy does not equate to severe end organ injury or vice versa, as previously illustrated (Figure 5). Furthermore, the strong correlation implies a fixed ratio between serum and cerebral lactate. It is unclear if this finding represents an equilibrium between systemic and cerebral lactate. Again, the current study was not designed to determine direction of lactate flux. Future studies that make use of carbon tagging are needed to further elucidate the direction of cerebral lactate flux in the infants with moderate–severe brain injury.

There are some limitations to the study. We were unable to assess the relationship between serum lactate and cerebral lactate after rewarming. Serum lactate values have often normalized by then and no further clinical trending of lactate values was warranted. Also, we were only able to transport clinically stable infants to the MRI suite during TH. Hence, the study cohort is subject to selection bias toward a moderate disease severity. Indeed, 39 out of the 48 infants were moderately encephalopathic on Sarnat exam.

## Conclusion

Serum lactate was elevated on admission and then normalized over time. The temporal evolution of cerebral lactate differed by injury severity. Serum lactate correlated modestly with cerebral lactate measured from the deep gray nuclei (thalamus and BG). Further studies are needed to study the effects of altered lactate metabolism in neonatal HIE.

## Ethics Statement

The research study was approved by the institution review board of the Human Subjects Protection Program at the Children’s Hospital Los Angeles. Written consent from the parent(s) were obtained by the researchers prior to all subject enrollment.

## Author Contributions

T-WW designed the research project, enrolled subjects, collected and analyzed data, and constructed the manuscript. K-HH collected and analyzed the data and helped with the improvement of the manuscript. AR was instrumental in subject recruitment, data analysis, and the proofreading of the manuscript. BT is a neuroradiologist who scored all the MR images according to the Barkovich scoring system for neonatal asphyxia. She also contributed to modification of the manuscript, specifically the Section “Materials and Methods.” MB was involved in the organization of MRS data, in designing of this study, and proofreading of the manuscript. EH was instrumental in subject recruitment, composition of the manuscript. SB was involved in the design of the research, collected, analyzed the data, and was instrumental in MR spectroscopy analysis. JW designed the research, contributed to generation of hypothesis and subsequent methodology, collected and analyzed data, and improved the manuscript.

## Conflict of Interest Statement

The authors declare that the research was conducted in the absence of any commercial or financial relationships that could be construed as a potential conflict of interest.
